# The C-Terminal SynMuv/DdDUF926 Domain Regulates the Function of the N-Terminal Domain of DdNKAP

**DOI:** 10.1371/journal.pone.0168617

**Published:** 2016-12-20

**Authors:** Bhagyashri D. Burgute, Vivek S. Peche, Rolf Müller, Jan Matthias, Berthold Gaßen, Ludwig Eichinger, Gernot Glöckner, Angelika A. Noegel

**Affiliations:** 1 Institute of Biochemistry I, Medical Faculty, Center for Molecular Medicine Cologne (CMMC), Cologne Excellence Cluster on Cellular Stress Responses in Aging-associated Diseases (CECAD), University of Cologne, Cologne, Germany; 2 Leibniz-Institute of Freshwater Ecology and Inland Fisheries, IGB, Berlin, Germany; Université de Genève, SWITZERLAND

## Abstract

NKAP (NF-κB activating protein) is a highly conserved SR (serine/arginine-rich) protein involved in transcriptional control and splicing in mammals. We identified DdNKAP, the *Dictyostelium discoideum* ortholog of mammalian NKAP, as interacting partner of the nuclear envelope protein SUN-1. DdNKAP harbors a number of basic RDR/RDRS repeats in its N-terminal domain and the SynMuv/DUF926 domain at its C-terminus. We describe a novel and direct interaction between DdNKAP and Prp19 (Pre mRNA processing factor 19) which might be relevant for the observed DdNKAP ubiquitination. Genome wide analysis using cross-linking immunoprecipitation-high-throughput sequencing (CLIP-seq) revealed DdNKAP association with intergenic regions, exons, introns and non-coding RNAs. Ectopic expression of DdNKAP and its domains affects several developmental aspects like stream formation, aggregation, and chemotaxis. We conclude that DdNKAP is a multifunctional protein, which might influence *Dictyostelium* development through its interaction with RNA and RNA binding proteins. Mutants overexpressing full length DdNKAP and the N-terminal domain alone (DdN-NKAP) showed opposite phenotypes in development and opposite expression profiles of several genes and rRNAs. The observed interaction between DdN-NKAP and the DdDUF926 domain indicates that the DdDUF926 domain acts as negative regulator of the N-terminus.

## Introduction

SR proteins are a conserved family of proteins comprising long repeats of serine (S) and arginine (R) amino acid residues, known as RS domain, important for protein localization to nuclear speckles. NKAP is an RS domain containing protein and was first identified as an activator of NFκB, making it a component of NFκB signaling [1]. NKAP comprises a tripartite domain architecture, the N-terminal RS domain followed by a basic domain and the C-terminal DUF926 (domain of unknown function). The C-terminal DUF926 domain was later classified as SynMuv based on the vulvar development phenotype in *Caenorhabditis elegans* [2]. In mammals, the DUF926 domain was shown to have a controlling function for NKAP [3]. NKAP associates with HDAC3 via its DUF926 domain and was shown as transcriptional regulator required for T cell development [4] and in the maintenance and survival of hematopoietic stem cells [5]. A recent report identified NKAP as a member of ~100 non-core spliceosomal proteins present in lower abundance at the spliceosome [6]. We confirmed the role of NKAP in RNA splicing and processing through direct association with RNA and RNA binding proteins [3]. Moreover, MAS2 (Morphology of Ago1-52 suppressed), the *Arabidopsis thaliana* ortholog of human NKAP, interacts with splicing and ribosome biogenesis proteins. MAS2 is an essential gene whose null alleles are embryonic lethal [7].

In *Drosophila melanogaster*, *Drosophila* NKAP ortholog CG6066 showed an interaction with three uncharacterized binding proteins which subsequently were linked to splicing components [8]. For mammalian NKAP, HDAC3, CBF1 interacting corepressor (CIR), RNA binding proteins, RNA helicases and splicing factors have been described as potential interaction partners [3,9]. Therefore, NKAP in addition to its role as transcriptional repressor appears to have a role in RNA splicing and RNA biogenesis [3,7].

*Dictyostelium discoideum* is a unicellular soil-living organism that upon starvation transitions into multicellularity, and thus is a model organism for studying chemotaxis, aggregation and development. The *D*. *discoideum* genome contains ~12,500 genes which are packed into six chromosomes. In general, *D*. *discoideum* pre-mRNAs contain few and short introns with an average size of 146 bp. Out of all protein-coding genes, 69% are spliced with an average of 1.9 introns per gene [10], indicating that pre-mRNA splicing is an essential process in *D*. *discoideum*. Spliceosomal RNAs (snRNAs) and proteins (splicing factors) have an important role in pre-mRNA splicing. In a large sequence survey using dictybase (http://dictybase.org/index.html) high sequence similarity was found between human and *D*. *discoideum* spliceosomal proteins including SR proteins [11]. The RS domains in *D*. *discoideum* SR proteins differ from those found in mammals as they are more enriched in the RDR/RDRS motif rather than in the typical RS/SR sequences found in mammalian SR proteins [11]. The major spliceosomal RNAs have been recognized in *D*. *discoideum*. Moreover they have all sequence motifs required for splicing and conserved secondary structure [12]. Apart from the six chromosomes, the *D*. *discoideum* genome contains rDNA palindromes that are localized on extrachromosomal elements surrounding nucleoli [13]. Although several precursor rRNA molecules are transcribed, mature rRNAs of the same type do not exhibit any diversity [13]. Several ncRNAs have been identified but the processing of ncRNAs and the characterization of RNA binding proteins remains elusive in *D*. *discoideum*.

Here we characterize the first SR protein in *D*. *discoideum* namely DdNKAP, ortholog of mammalian NKAP. DdNKAP carries RDR/RDRS motifs at its N terminus followed by a SynMuv/DdDUF926. We show that it localizes to the nucleus in a punctate pattern and that the DdBasic domain is important for the punctate pattern of localization. DdNKAP interacts with RNA binding proteins such as Prp19 and ribosomal proteins. CLIP-seq revealed that it interacts with exons, introns and ncRNAs. We complemented these studies with RNAseq and developmental analysis and demonstrate that the SynMuv/DdDUF926 domain has an impact on DdNKAP function.

## Material and methods

### Generation of GFP-DdNKAP fusion vectors and DdNKAP antibodies

The coding sequence of full-length DdNKAP and truncated proteins was amplified as a HindIII—BamHI fragment and cloned into vector pDex79 [14] and two independent transformations were done into AX2 cells. GFP was fused to the N-terminus of DdNKAP and truncated versions of DdNKAP and the fusion proteins were expressed under the control of the actin 15 promoter. Transformants were selected using G418. These transformants were plated onto a Klebsiella lawn and individual colonies were transferred into 96 well plates. Fusion protein expressing cells were identified under a fluorescence microscope and two clones were isolated from each transformation. Green fluorescent cells were fixed and the localization of the proteins analyzed. For the generation of DdNKAP specific antibodies, the C-terminal GST tagged DUF926 domain was used. Expression was in *E*. *coli* BL21, and induction was with isopropyl β-D-1-thiogalactopyranoside (1 mM). The GST tagged protein was purified using affinity chromatography with Glutathione-Sepharose beads and 50 μg of protein was used to immunize four female BALB/c mice, followed by four boosts of 50 μg each over 4 weeks. Hybridoma cells were generated as described [15]. The obtained mAb K79-232-2 was used in this study.

Animals were housed in the facility of the Medical Faculty, University Hospital, University of Cologne. Animal protocols were approved by the local veterinary authorities. Experiments were performed according to institutional guidelines and animal license 84–02.05.30.13.037 of the State Office of North Rhine-Westphalia, Germany. The study was approved by the Landesamt für Natur-, Umwelt- und Verbraucherschutz Nordrhein-Westfalen. Mice were obtained from Charles River Laboratories, Sulzfeld, Germany. They were killed by cervical translocation.

### Mutant analysis

For growth in liquid nutrient medium log phase AX2 wild type cells and DdNKAP overexpressors were inoculated in an equal volume of axenic medium at a density of 2 x 10^5^ cells/ml and grown at 23°C with shaking at 160 rpm [16]. Cells were counted at different time points after inoculation using a Neubauer chamber. The experiments were carried out in triplicates. To analyze the aggregation behavior, cells were grown in axenic medium in shaking suspension. At a density of 3 x 10^6^ cells/ml the cells were harvested and washed twice in ice-cold Soerensen phosphate buffer (17 mM Na^+^/K^+^ phosphate, ph 6.0). The cells were again reconstituted at a density of 1 x 10^7^ cells/ml in Soerensen phosphate buffer and plated as monolayers on plastic petri dishes (1 x 10^5^ cells/cm^2^). At different time points during aggregation images were captured using an Olympus IX70 inverse microscope. For development on a solid substratum, 5 x 10^7^ cells were plated onto 10 cm phosphate agar plates (Soerensen phosphate buffer). Incubation was at 23°C. Phototaxis analysis was done as described [16]. The growth, aggregation experiments were performed with two independent transformants.

### Chemotaxis assay and video imaging

Cells were starved for 5 to 6 h in Soerensen phosphate buffer (23°C, 160 rpm) at a density of 1 x 10^7^ cells/ml. 25–30 μl of the cell suspension was diluted in 3 ml of Soerensen phosphate buffer and mixed well by pipetting (25–30 times, with occasional vortexing). This is important in order to dissociate aggregates. 1.5 ml of the diluted cells were then transferred onto a glass cover-slip with a plastic ring placed on a Leica inverse microscope equipped with a 20x UplanFl 0.3 objective. Cells were stimulated with a glass capillary micropipette (Eppendorf Femtotip) filled with 0.1 mM cAMP [17], which was attached to a microcontroller. Time-lapse images were captured at 30 seconds intervals with a JAI CV-M10 CCD camera and an Imagenation PX610 frame grabber (Imagenation Corp., Beaverton, OR) controlled through Optimas software (Optimas Corp., Bothell, Washington) and stored on a computer hard drive.

### Immunofluorescence analysis and protein-protein interaction studies

To observe the localization of GFP-DdNKAP during cytokinesis, cells were first seeded on glass coverslips and synchronized using nocodazole (10 μM) for 3 h to block cell division in mitosis. The block was then released by washing away the drug and allowing the cell cycle to progress for 1 h. Cells were fixed with ice cold methanol. For α-tubulin staining rat mAb YL1/2 [18] was used to identify mitotic cells. DNA was stained with DAPI (49, 6-diamidino-2-phenylindole, Sigma).

Immunoprecipitation of GFP-DdNKAP was performed using 20 μl GFP-Trap_ A (agarose) beads (ChromoTek, Martinsried, Germany) from extracts of cells (1 x 10^7^) expressing GFP and GFP-DdNKAP. The lysis buffer contained 50 mM Tris-HCl, pH 7.4, 100 mM NaCl, 1% NP 40, 0.1% SDS, 0.5% sodium deoxycholate, 1 mM PMSF and protease inhibitors (Sigma). The beads were washed three times with 500 μl lysis buffer and boiled in 1x sample buffer (20 μl) which were resolved by SDS-PAGE and immunoblotted with monoclonal antibodies against Sun-1 (K55-432-2) [19] and GFP (K3-184-2) [16].

The interaction between DdNKAP and Prp19 was studied through GST pull-down assays. Full-length and truncated versions of GST-Prp19 proteins were produced in *E*. *coli* Arctic cells. Cells were lysed (50 mM Tris-HCl, pH 8, 300 mM NaCl, 0.05% NP 40, 1 mM PMSF and protease inhibitors (Sigma)) and GST-fusion proteins were bound to Glutathione-Sepharose 4B beads (GE Healthcare). Beads containing GST or beads only were used as a control. AX2 cells (1 x 10^7^) expressing GFP-DdNKAP, GFP-DdRS, GFP-DdBasic or GFP-DdDUF926 were suspended in 500 μl of lysis buffer (50 mM Tris-HCl, pH 7.4, 100 mM NaCl, 1% NP 40, 0.1% SDS, 0.5% sodium deoxycholate, 1 mM PMSF and protease inhibitors (Sigma)). The extract was centrifuged at 14,000 r.p.m. for 10 min. The supernatant was incubated with 20 μl of beads coupled with GST and GST fusion proteins for 2 hours. Then beads were washed three times with 500 μl washing buffer (50 mM Tris-HCl, pH 7.4, 100 mM NaCl) and boiled with 1x SDS sample buffer (20 μl). The proteins were resolved by SDS-PAGE (12% acrylamide) and immunoblotted with GFP antibodies (mAb K3-184-2).

Ubiquitination was studied through immunoprecipitation. GFP-Trap beads were used to pull down GFP-DdNKAP and GFP alone from 500 μl extracts of cells (1 x 10^7^) expressing GFP and GFP-DdNKAP prepared as described above. Beads were washed three times with 500 μl lysis buffer and boiled with 1x SDS sample buffer. The proteins were subsequently resolved by SDS-PAGE (12% acrylamide) and immunoblotted with the anti-ubiquitin mAb P4D1 (cell signaling technology) and anti GFP mAb K3-184-2.

For the direct DdNKAP and Prp19 interaction, GST-Prp19 polypeptides were incubated for 2 hours with DdRS, DdBasic and DdDUF domains released from GST through thrombin cleavage. Afterwards beads were washed three times with washing buffer (50 mM Tris-HCl, pH 7.4, 50 mM NaCl). The pulldown samples were boiled with 1x SDS sample buffer (20 μl) and the proteins resolved by SDS-PAGE (15% acrylamide) and stained with Coomassie Blue.

### RNAseq and Microarray Analysis

The RNAseq experiments were performed with RNA from AX2 and GFP-DdNKAP expressing AX2 cells from three biological replicates. For library preparation, the TruSeq stranded mRNA library prep kit (Illumina) was used. First, 1 μg of each total RNA sample was used for polyA mRNA selection using streptavidin-coated magnetic beads. The polyA selected mRNA was fragmented and amplified for cDNA synthesis using reverse transcriptase and random hexamer priming. In addition, the amplified cDNA underwent double stranded cDNA conversion, end repair and adaptor ligation process. Size selection was performed using gel purification (2% agarose gel) generating cDNA libraries ranging in size from 200–250 bp. Finally, the libraries were amplified using PCR (15 cycles) and quantified using Bioanalyzer 2100 (Agilent). Each library was run at a concentration of 7 pmol using paired-end 75 bp sequencing on Hiseq 4000 device (Illumina).

The RNA-seq raw reads were aligned to the *D*. *discoideum* genome sequence (http://dictybase.org/) using default values with Bowtie2 [20]. Using the DEseq package significantly differentially expressed genes were extracted [21]. GO (gene ontology) analysis was performed using the PANTHER Classification system, version 11.0 [22] and DAVID Bioinformatics Resources 6.8 [23].

For microarray analysis, in total six microarrays were analysed with labelled cDNAs derived from three independent RNA isolations from AX2 wild-type cells (control) and GFP-DdNKAP expressing AX2 cells (experiment). Microarray analysis was essentially carried out as described [24]. Briefly, fluorescence ratios were normalised by LOWESS-normalisation and differentially expressed genes were identified with the program Significance Analysis of Microarrays (SAM) [25]. The SAM program not only identifies the differentially regulated genes, but also predicts the number of false positives (FDR = False Discovery Rate). Our analysis was performed with the adjustment that the 90th percentile of the FDR was minimal. Of the reported genes only those were further analysed that were in addition at least 1.5-fold differentially expressed. For qRT-PCR, reactions were run in quadruplet in three independent experiments. The geometric mean of the housekeeping gene GAPDH was used as an internal control to normalize the variability in expression levels. The primer sequences are provided in S1 Table. Expression data were normalized to the housekeeping gene GAPDH.

DGAP1 and cortexillin I expression levels were analyzed by western blot analysis. Lysates of AX2 and GFP-DdNKAP expressing AX2 were resolved by SDS-PAGE and immunoblotted with DGAP1 and cortexillin I antibodies [26].

### CLIPseq analysis

CLIP-seq was performed as described previously [27]. Briefly, AX2 cells expressing GFP-DdNKAP or GFP alone were cultivated in 15-cm dishes and irradiated at 300mJ/cm^2^ UV light using a UVC500 UV chamber (Hoefer). The cell lysis was carried out in 1 ml lysis buffer (50 mM Tris-HCl, pH7.4, 100 mM NaCl, 1% NP-40, 0.1% SDS, 0.5% sodium deoxycholate, protease inhibitor cocktail (Sigma)). The lysates were incubated with 1U, 2U and 20U of RNase T1 and 2 μl Turbo DNAase for 3 min at 37°C. The lysates were centrifuged (16000 r.p.m. for 15 min) and supernatant was incubated with GFP-Trap beads for 2 h at 4°C. The beads were washed 2x with high salt buffer (50 mM Tris-HCl, pH 7.4, 1 M NaCl, 1 mM EDTA, 1% NP-40, 0.1% SDS, 0.5% sodium deoxycholate) and 2x with wash buffer (20 mM tris-HCl, pH 7.4, 10 mM MgCl_2_, 0.2% Tween-20). The beads were incubated in 8 μl of hot polynucleotide kinase (PNK) mix (0.4 μl PNK, 0.8 μl 32P-ATP, 0.8 μl 10x PNK buffer, 6 μl water) for 5 min at 37°C. The PNK mix was removed and the beads were resuspended in 20 μl 1x Nupage loading buffer (Invitrogen) and incubated at 70°C for 10 min. The RNA-protein complex was separated on NuPAGE Bis-Tris gel (Invitrogen), transferred to a nitrocellulose membrane and an autoradiograph was obtained. The RNA was isolated from nitrocellulose membrane by incubating the membrane at 37°C for 20 min in PK buffer (100 mM Tris-HCl pH 7.4, 50 mM NaCl, 10 mM EDTA, 7 M urea, proteinase K (0.2 mg/ml)). The RNA was extracted by phenol chloroform extraction and ethanol precipitation, which was used for library preparation. The library preparation and sequencing were performed at the CCG (Cologne Center for Genomics). The CLIP raw reads were aligned to the *D*. *discoideum* genome sequence (http://dictybase.org/) using Bowtie2 [20]. We counted reads associated with genomic features with the tophat2 program [28]. For peak finding we used Piranha (http://smithlabresearch.org/software/piranha/).

## Results

### The domain architecture of DdNKAP is conserved

**Fig 1:**
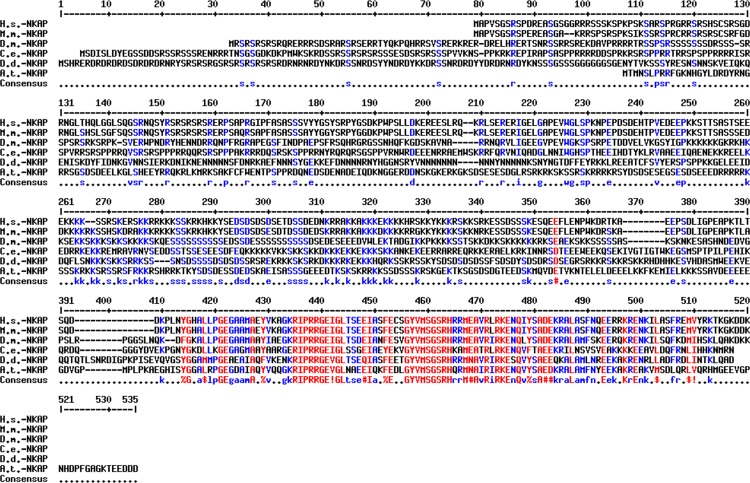
Sequence conservation among eukaryotic NKAP proteins. Sequence alignment of NKAP from different species using the multalin sequence alignment. Abbreviations: H.s.: *Homo sapiens*; M.m.: *Mus musculus*; D.m.: *Drosophila melanogaster*; C.e.: *Caenorhabditis elegans*; A.th.: *Arabidopsis thaliana*; D.d.: *Dictyostelium discoideum*. Sequence IDs are as follows: *H*.*s*. (NP_078804), *M*.*m*. (NP_080213), *D*.*m*. (AAF56669), *C*.*e*. (CCD68576), *A*.*th*. (AT4G02720) and *D*.*d*. (XP_645847). Highly conserved amino acids are shown in red, less conserved in blue and and neutral ones in black, respectively. Consensus symbols are “!”, I or V; $, L or M; %, for Y; #, any one of N, D, Q, E. B.

**Fig 2:**
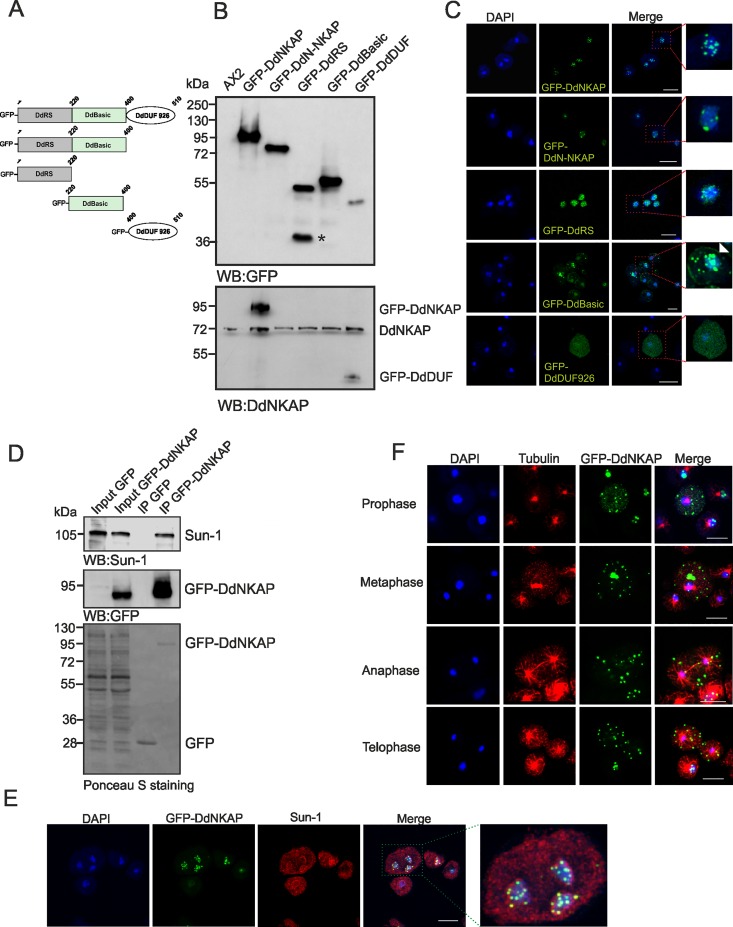
Localization of full-length and truncated versions of GFP-DdNKAP. (A) The figure depicts the constructs used in the analysis. The DdRS domain, the basic domain and the DUF926 domain of DdNKAP are shown schematically. (B) Lysates of AX2 and of full-length and truncated versions of GFP-DdNKAP expressing cells were immunoblotted and probed using mAb K3-184-2 for GFP and mAb K79-232-2 for DdNKAP. The asterisk marks a degradation product of GFP-DdRS. (C) Full length DdNKAP, the DdN-NKAP, DdRS, DdBasic and DdDUF domains were tagged with N-terminal GFP and expressed in AX2 cells that were fixed and stained with DAPI. Scale bar, 10 μm. The red frames in the right images mark the regions which are shown at higher magnifications on the right. (D) Co-immunoprecipitation of DdNKAP and SUN1. Immunoprecipitation was performed using GFP-Trap beads followed by immunoblotting with mAb K55-432-2 (Sun-1) and mAb K3-184-2 (GFP). The Ponceau S stained membrane is shown at the bottom. (E) AX2 cells expressing GFP-DdNKAP were fixed with methanol and labeled with mAb K55-432-2 for Sun-1. DNA was stained with DAPI. Scale bar, 10 μm. (F) Cells expressing GFP-DdNKAP (green) were synchronized using nocodazole to block progression of the cell cycle and then released and fixed using cold methanol. Tubulin staining (red) using rat mAb YL1/2 was used to identify mitotic cells. Nuclei (blue) were stained with DAPI. Bar, 10 μm.

Previously, we characterized the nuclear envelope protein Sun-1 which is a component of the LINC complex and can connect the centrosome to chromatin to ensure genome stability [19,29]. To further study the function of Sun-1, we performed co-IP experiments followed by LCMS (Liquid chromatography-mass spectrometry) analysis. The interacting partners were identified (S2 Table), among them DdNKAP (*D*. *discoideum* NF Kappa B Activating Protein, DDB0306239, DDB_G0269284), an ortholog of mammalian NKAP. DdNKAP is a 510 amino acid residues containing protein which can be divided into two domains. The N-terminal domain is composed of highly basic amino acids containing segments of low compositional complexity; the C-terminal domain has a SynMuv/DdDUF926 domain. The N-terminal domain contains RDR and RS repeats, which is a signature of SR proteins in *D*. *discoideum* [11], and can be subdivided into a RS-domain (aa 1–220) followed by a basic domain (aa 221–400). The DdDUF926 domain (aa 401–510) is highly conserved and shows ~70% identity with the one in mammalian NKAP. NKAP of *D*. *melanogaster*, *Caenorhabditis elegans* and *D*. *discoideum* harbour a unique extended N-terminus as compared to mammalian NKAP (Figs 1 and 2A).

### DdNKAP is localized in the nucleus in a punctated pattern

In order to test the interaction of DdNKAP with Sun-1 and to study its subcellular localization, we expressed full length DdNKAP (aa 1–510), DdN-NKAP (aa 1–400), DdRS (aa 1–220), DdBasic (aa 221–400) and DdDUF (aa 401–510) as GFP fusion proteins in AX2 wild-type cells (Fig 2A). The expression of these domains was probed by western blot analysis using GFP (mAb K3-184-2) and DdNKAP (mAb K79-232-2) antibodies. As the DdNKAP antibodies are directed against the DdDUF926 domain, it recognizes GFP-DdNKAP and GFP-DdDUF926 domain only (Fig 2B). GFP-DdNKAP localized exclusively in the nucleus and exhibited a punctate pattern similar to mammalian NKAP [3]. The DdN-NKAP fusion protein localized in the nucleus in a punctate pattern similar to the full length protein (Fig 2C). DdRS was exclusively localized in puncta and also diffusely distributed throughout the nucleus. The DdBasic domain showed punctate nuclear and at times cytosolic localization. Occasionally DdBasic was present at the cell cortex as well (Fig 2C, arrowhead in magnification). In contrast, DdDUF926 was diffusely present throughout the nucleus and the cytoplasm (Fig 2C). Furthermore, nuclear and cytosolic fractionation confirmed the nuclear localization of DdNKAP, DdN-NKAP and DdRS domain. The DdBasic and DdDUF domains were present in the nuclear as well as the cytosolic fraction (S1A Fig).

Next we examined the DdNKAP-Sun1 interaction by using GFP-DdNKAP for immunoprecipitation. GFP-DdNKAP could immunoprecipitate endogenous Sun-1 whereas GFP alone for control did not show any interaction (Fig 2D). This was also seen in immunofluorescence studies, where DdNKAP partially colocalized with Sun-1 (Fig 2E). In mammals, nuclear speckles are situated in interchromatin regions also known as interchromatin granule clusters. Neither proteins localizing to nuclear speckles of *D*. *discoideum* are well studied nor is their distribution and appearance known. Nevertheless we observed DdNKAP punctae in close proximity of the nuclear envelope, which explains colocalization with Sun-1.

As *D*. *discoideum* mitosis is a semi-open process [30], we further studied the localization of DdNKAP during mitosis. AX2 cells expressing GFP-DdNKAP were fixed after release from a nocodazole block. Interestingly, unlike previous reports where nuclear proteins were diffusely distributed throughout the cytoplasm during mitosis [31–33], DdNKAP remained in a punctate pattern in the cytoplasm throughout mitosis (Fig 2F). This was reminiscent of mammalian nuclear speckle proteins that are distributed in the cytoplasm during mitosis and are associated with mitotic interchromatin granules (MIGs) [34].

### DdNKAP interacts with RNA binding and ribosomal proteins

**Fig 3:**
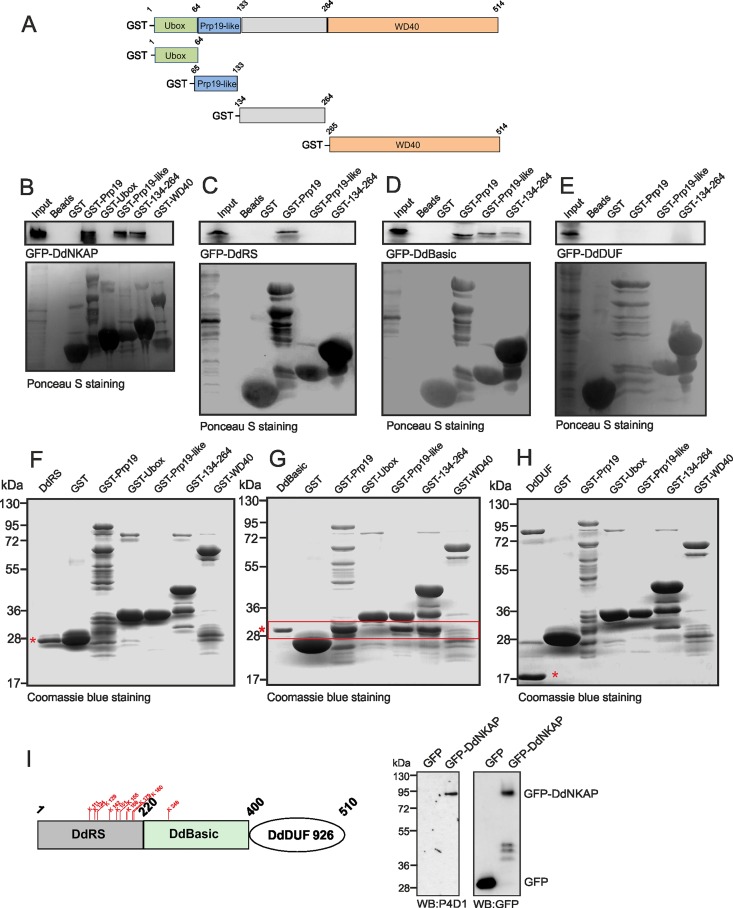
Interaction of DdNKAP and Prp19. (A) Schematic representation of the Prp19 domain structure and Prp19 constructs used in this study. (B, C, D, E) GST tagged full length Prp19, the Prp19-like and the 134–264 domains pull down GFP-DdNKAP and GFP-DdBasic recognized by mAb K184-3. GST-Prp19 polypeptides and GST were visualized by Ponceau S staining. Proteins were separated by SDS-PAGE (12% acrylamide). The GFP-DdBasic domain interacts with GST-Prp19, GST-Prp19-like and GST-134-264. (F, G, H) The GST tagged full length Prp19 and truncated Prp19 proteins were incubated with bacterially expressed, purified and thrombin cleaved DdRS (F), DdBasic (G) and DdDUF (H). Beads were washed and proteins were separated by SDS-PAGE (15% acrylamide) and visualized by Coomassie Blue staining. DdRS, DdBasic and DdDUF are marked with red asterisks; the red box denotes the direct interaction of DdBasic with GST tagged full length Prp19, Prp19-like and 134–264. (I) The DdRS domain carries several predicted potential ubiquitination sites (indicated in red). GFP and GFP-DdNKAP were immunoprecipitated by GFP-Trap beads. The ubiquitinated DdNKAP was detected by western blot with anti-ubiquitin P4D1 mAb and the immunoprecipitation of GFP-DdNKAP and GFP was monitored by mAb K3-184-2.

To unravel the function of DdNKAP we next tried to identify further binding partners. We searched for interacting proteins of DdNKAP using a co-immunoprecipitation approach. Interestingly, we found eight RNA binding proteins and several ribosomal subunit proteins (S3 Table). Among the RNA binding proteins we focused on Prp19 (Pre mRNA processing factor 19). Prp19 is extensively studied in yeast and mammals. It functions in several cellular processes. It is a core component of the Prp19 complex also known as NineTeen Complex (NTC) which is a part of the spliceosome and participates in assembly and remodeling of the spliceosome during splicing [35]. Furthermore, it is a ubiquitin protein ligase and thought to be involved in protein degradation [35]. It contains an N-terminal Ubox domain (Ring superfamily) followed by a Prp19-like domain, an uncharacterized region and six C-terminal WD40 repeats (Fig 3A). *D*. *discoideum* Prp19 shows 37% identity and 57% homology with mammalian Prp19.

We used GST fusion proteins with full length and truncated Prp19 in pulldown assays in order to test the interaction with DdNKAP and to identify interacting domains. GST tagged Prp19 could efficiently pull down GFP-DdNKAP. We found that the Prp19-like (aa 64–133) and the neighboring region encompassing amino acids 134–264 did bring down GFP-DdNKAP (Fig 3B). Furthermore, we narrowed down the association site in DdNKAP using different DdNKAP polypeptides fused to GFP. GST-Prp19, GST-Prp19-like and GST-134-264 were used in the pull downs. The DdRS domain interacted only with full length Prp19 (Fig 3C), the DdBasic domain interacted with full length Prp19, Prp19-like and the 134–264 polypeptide (Fig 3D) and the DdDUF domain did not show any interaction (Fig 3E). In a GST-pulldown assay, the interaction could be direct or indirect as there is the possibility that this interaction is mediated through unknown cellular protein. However, the direct binding assay ensures the direct physical interaction between two proteins as both proteins are purified and tested further by immobilizing one on beads and employing the other to check the direct binding capacity. To address the direct interaction and to determine which protein domains of DdNKAP and Prp19 are necessary for the interaction, we used bacterially expressed GST tagged Prp19 domains as bait to pull down purified DdNKAP domains. For the DdRS domain we did not observe a direct interaction with Prp19 or its domains, although GST-Prp19 precipitated GFP-DdRS (Fig 3C and 3F). GST-Prp19, GST-Prp19-like and GST-134-264 could successfully pulldown the DdBasic domain of DdNKAP (Fig 3G, red rectangle), further we did not observe direct interaction between Prp 19 and DdDUF (Fig 3H). Taken together, these data indicate that the DdBasic domain of NKAP is responsible for a direct interaction and that it preferentially interacts with the Prp19-like domain and a domain containing amino acids 134–264 of Prp19.

Since Prp19 is a member of the Ubox family of E3 ubiquitin ligases, the significance of the DdNKAP and Prp19 interaction could be ubiquitination of DdNKAP. To check this possibility, we searched for ubiquitination sites in DdNKAP and found several high confidence ubiquitination sites in the DdRS and DdBasic domains of DdNKAP. Further, we performed immunoprecipitation by utilizing GFP-DdNKAP and GFP expressing AX2 cells. The immunoprecipitation was followed by immunoblotting with the P4D1 mAb recognizing ubiquitinated proteins and with GFP specific antibodies. When the blot was probed with P4D1 antibodies, the ubiqutinated form of DdNKAP appeared whereas no signal was detected in case of GFP which was used as control. The membrane was then stripped and probed with anti-GFP antibodies to confirm the successful precipitation of GFP-DdNKAP and GFP proteins (Fig 3I).

### DdNKAP is an RNA binding protein

**Fig 4:**
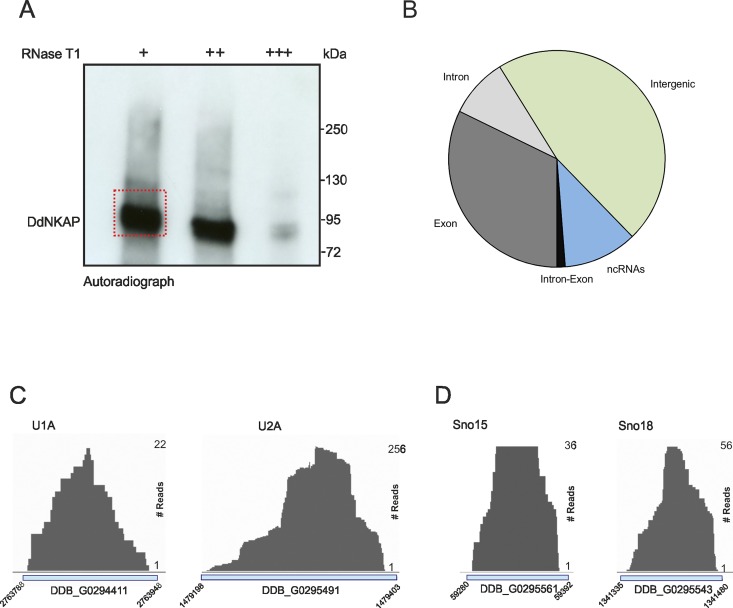
Global analysis of DdNKAP binding to RNA by CLIPseq. (A) Autoradiograph of cross-linked DdNKAP-RNA complexes using denaturing gel electrophoresis and membrane transfer. RNA was partially digested using increasing concentrations of RNase T1. The marked area was cut from the membrane and subjected to protocols for the isolation of RNA, which was further used for RNA-seq. Positions of proteins from the molecular mass standard (kDa) are shown on the right. (B) Percentage of uniquely identified genes in CLIPseq with their respective genomic features. CLIP tags are mapped to exons, introns, intergenic regions and non-coding RNAs according to the gene predictions in dictyBase (http://dictybase.org/). (C,D) DdNKAP association with U1, U2, sno15 and sno18. The dictybase gene ID and coordinates are mentioned at the x-axis of each plot. CLIP-seq reads are mentioned at the right side of each plot.

NKAP, the mammalian ortholog of DdNKAP, interacts with RNA and plays a role in RNA biogenesis. We therefore checked the possibility of a DdNKAP-RNA interaction through CLIP and generated a genome wide map of DdNKAP-RNA interactions *in vivo*. Since our antibodies do not immunoprecipitate DdNKAP we used UV irradiation to crosslink protein-RNA complexes in AX2 cells expressing GFP-DdNKAP and immunoprecipiated the complexes with GFP antibodies (Fig 4A). As control cross-linked and non-cross-linked AX2 cells expressing GFP were used, which produced no signal (S1B Fig). Isolated RNA fragments were sequenced by CLIPseq, the resulting reads processed and aligned to the *D*. *discoideum* genome. The experiment was performed in four biological replicates. In total 67,007,478 reads were mapped to the genome (S4 Table). We found that the majority of CLIP tags were located within rRNAs, intergenic and exonic regions according to position data of genomic features obtained from the gff-file downloaded from dictybase (http://dictybase.org/). DdNKAP reads were also identified within introns, ncRNAs and intron-exon junction (Fig 4B) (S5 Table). The occurrence of reads at intron-exon borders was rather low. This could be due to the fact that *D*. *discoideum* genes contain few and short introns.

The most abundant ncRNA classes in the CLIP tags were rRNAs. Piranha analysis confirmed that the CLIP experiments resulted in strong peaks on ncRNAs and importantly with rRNAs. These peaks were called as statistically significant using a zero-truncated negative binomial distribution analysis. Furthermore, we found DdNKAP binding with U1 and U2 snRNAs which have a crucial role in pre-mRNA splicing (Fig 4C). Interestingly, we also found sno15 and sno18 which are involved in rRNA biogenesis. Moreover, we found signal recognition particle RNAs srpA and srpB, which are members of a family of small non ribosomal RNAs localized to the nucleolus.

### Comparison of the transcriptional expression profiles of vegetatively growing AX2 and GFP-DdNKAP expressing cells

**Fig 5:**
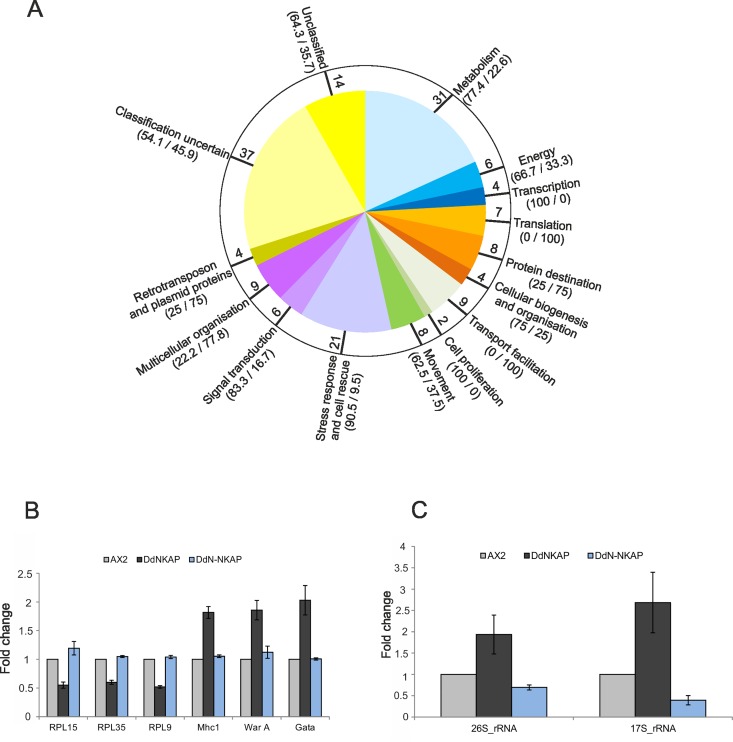
Transcriptional profile of differentially regulated genes in DdNKAP overexpressors. (A) Pie diagram showing the functional classification according to the yeast classification scheme of the differentially regulated genes. The values in the bracket indicate the percentage of up- and down-regulated genes, respectively, in GFP-DdNKAP expressing cells in comparison to AX2. The values inside the circle indicate the total number of differentially regulated genes in the respective processes. (B) Confirmation of the differential regulation of selected genes by qRT-PCR. Three down-regulated (RPL15, RPL35, RPL9) and three-upregulated (Mhc1, WarA, Gata) genes were chosen to confirm the microarray data. The experiment was performed in technical quadruplets and biological triplicates. (C) Analysis of rRNA expression in DdNKAP mutants by qRT-PCR. Three biological replicates with four technical replicates each were performed.

Having established that DdNKAP associates with a spliceosome associated protein and with RNA we wanted to find out whether overexpression would have an impact on the transcriptional profile. DNA microarrays and RNA-seq were used to compare the transcriptional profiles of vegetative (t0) cells of AX2 and AX2 harboring a GFP-DdNKAP construct. The microarray used carried cDNA sequences that represent approximately 50% of the encoded genes (Gene Expression Omnibus accession number GPL1972) [24]. We hybridized six microarrays with labelled cDNAs derived from three independent RNA isolations and used the program Significance Analysis of Microarrays (SAM) [25] to identify differentially expressed genes (Fig 5A). SAM reported 742 genes as significantly differentially regulated. However, more than 500 of these displayed only very low folds of change and therefore we further analyzed only those 170 genes which were in addition at least 1.5 fold differentially regulated. Out of these 170 genes, 100 were up- and 70 genes were down-regulated in the GFP-DdNKAP expressing cells compared to wild type AX2 cells (S6 Table). The differentially regulated genes were functionally categorized based on the yeast classification scheme which was adapted for *D*. *discoideum* [36]. This classification showed that upregulation occurred in the stress response and cell rescue category (90% of the differentially regulated genes were upregulated, 10% down). In contrast, translation was down-regulated (100% of differentially regulated genes). A considerable proportion of genes (~32%) that were significantly differentially regulated in the GFP-DdNKAP expressing cells belonged to the classification categories “uncertain” or “unclassified protein” (Fig 5A). We probed the microarray data by qRT-PCR for three upregulated genes and three downregulated genes and found that these genes were differentially regulated in the GFP-DdNKAP mutant confirming the microarray data. Interestingly, no difference in expression of these genes was observed in the GFP-DdN-NKAP mutant (Fig 5B). Moreover rRNA expression was upregulated in GFP-DdNKAP while it was down regulated in the GFP-DdN-NKAP mutant (Fig 5C) which indicates a regulatory role for the DdDUF926 domain. We further confirmed the microarray data by western blot analysis focusing on DGAP1 and cortexillin I. The result showed that DGAP1 and cortexillin I levels were significantly increased when compared to AX2 (S1C and S1D Fig).

**Fig 6:**
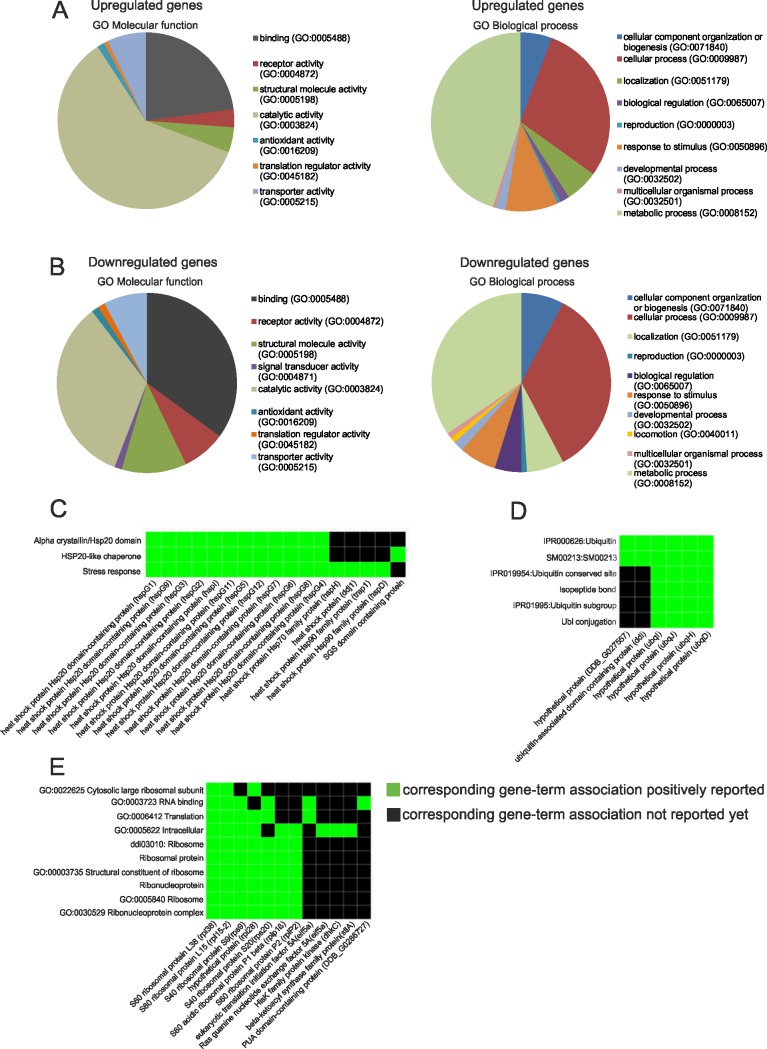
RNAseq analysis of DdNKAP overexpressors and AX2 cells using GO Molecular function and GO Biological process of differentially expressed genes. Upregulated (A) and downregulated (B) genes were classified for Gene ontology molecular function and biological process using the PANTHER classification system. (C, D, E) Upregulated and downregulated genes were applied to DAVID to identify functional ontological groups. Shown are specific heat-map examples: (C) Stress response, (D) Ubiquitin, (E) Ribosomal proteins. The heat-maps are derived from the report generated by DAVID, and are an annotated-term-focused view which lists annotated genes and their category. Green represents corresponding gene-term association positively reported. The black represents corresponding gene-term association not reported yet. All results passed the default thresholds, to ensure that only statistical significant groups are displayed.

To further probe the microarray data and to extend the expression analysis we used RNAseq. To this end we sequenced three independent samples (biological replicates) for both AX2 and GFP-DdNKAP expressing cells. We then calculated significantly differentially expressed genes using the DEseq package. This analysis identified 466 upregulated and 178 downregulated genes in the GFP-DdNKAP expressing cells compared to the AX2 wild type (S7 Table). The RNAseq differentially expressed genes were subjected to GO analysis using the PANTHER Classification System, version 11.0. PANTHER revealed that binding and catalytic activity in the molecular function classification and cellular and metabolic processes in the biological processes categorization were the main categories in the up- and down-regulated genes (Fig 6A and 6B). To further annotate and weigh these genes in functional classes, we additionally utilized the functional annotation clustering from DAVID Bioinformatics Resources 6.8. From our upregulated genes we were able to find two interesting functional classes with relevance to our studies, stress response and ubiquitin (Fig 6C and 6D) whereas a functional class in the downregulated genes was ribosomal proteins (Fig 6E).

48% of the upregulated and 22% of the downregulated genes from microarray experiments were found in the RNAseq data. Since the significance threshold of the DEseq analysis is higher and does not rely on absolute values for expression difference, this low accordance is not surprising. However, genes categorized in stress response and ribosomal proteins were found with both methods, suggesting that overexpression of DdNKAP directly or indirectly slows down translation and protein turnover.

### DdNKAP mutants show an extended lag phase in axenic growth

**Fig 7:**
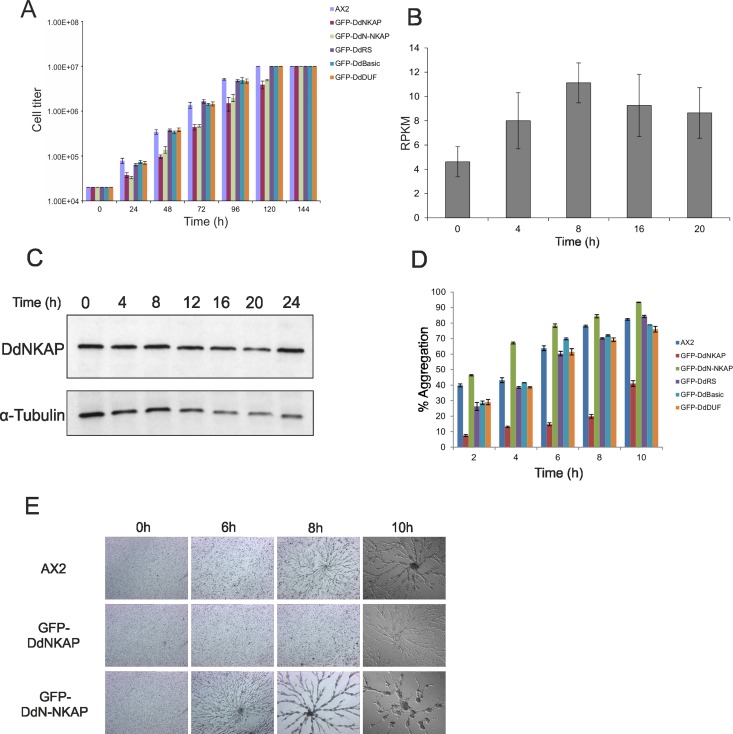
Growth and development of GFP-DdNKAP mutants. (A) Growth of AX2 and AX2 expressing GFP-DdNKAP mutants in shaking culture. The cell numbers were determined every 24 hours. Mean values and standard deviations of growth curves from three independent experiments are shown. (B) Expression profile of DdNKAP in vegetative and developing *D*. *discoideum* cells was extracted from dictyBase (http://dictybase.org/). Y-axis represents DdNKAP reads per kb per million mapped reads (RPKM), while the X-axis shows developmental time points (h). (C) Analysis of DdNKAP expression in AX2 using mAb K79-232-2and YL1/2 for tubulin as control. (D) AX2 and GFP-DdNKAP mutants were allowed to aggregate in Soerensen phosphate buffer in shaking suspension. Samples at different time points (2, 4, 6, 8, 10 h) during aggregation were taken and the aggregate formation (given in %) was scored by measuring the absorbance of the suspension at 600 nm. Mean values and standard deviations of growth curves from two independent experiments are shown. (E) Development in submerged culture. Cells (AX2, GFP-DdNKAP and GFP-DdN-NKAP) at equal densities (2 x 10^5^ cells/cm^2^) were starved submerged in Soerensen buffer on plastic petri dishes and monitored for aggregation. Images were taken every hour (10X magnification) until 10 hours of starvation. Images of the indicated time points are shown.

*D*. *discoideum* is a convenient model organism in which one can study cellular and developmental aspects [37]. To study the function of DdNKAP in *D*. *discoideum* we generated knockout and knock down vectors (S2A and S2B Fig). Since we were not successful in obtaining knockout or knockdown strains we resorted to the analysis of GFP-DdNKAP, GFP-DdN-NKAP, GFP-DdRS, GFP-DdBasic and GFP-DdDUF overexpressing AX2 cells to get information on processes in which DdNKAP is involved. The growth behavior of the strains was assayed in suspension culture in axenic medium. The GFP-DdNKAP and GFP-DdN-NKAP overexpressors appeared to grow slower than AX2 cells. A detailed examination showed that both mutant strains had an extended lag phase, however, once growth started, no major differences were observed in the doubling time. They reached the same final densities of 1 x 10^7^ cells/ml. GFP-DdRS, GFP-DdBasic and GFP-DdDUF expressing AX2 cells behaved like the parent strain (Fig 7A).

### DdNKAP overexpression leads to a delay in stream formation during multicellular development

To examine the DdNKAP expression during developmental stages the RPKM (reads per kb per million mapped reads) values of DdNKAP were extracted from dictybase revealing that DdNKAP is differentially expressed during development (Fig 7B). Further we assessed the protein levels of DdNKAP in growing and developing cells by western blot analysis which, by contrast, showed similar levels throughout development (Fig 7C). Based on this pattern we investigated the developmental behaviour of cells expressing the GFP tagged proteins which should be present in high amounts throughout development due to the constitutively active actin 15 promoter.

When no food source is available amoebae aggregate and develop into a fruiting body. To study the aggregation phase, we monitored aggregate formation during starvation in shaken suspension by following the decrease in OD600 and found that GFP-DdN-NKAP expressing cells formed aggregates earlier than AX2 whereas GFP-DdNKAP cells showed strongly reduced aggregate formation. GFP-DdRS, GFP-DdBasic and GFP-DdDUF did not show any significant difference in aggregate formation when compared to AX2 (Fig 7D).

As GFP-DdNKAP and GFP-DdN-NKAP expressors showed altered aggregation, we analyzed aggregation and stream formation under submerged conditions in a Petri dish. Major differences were observed with AX2 GFP-DdNKAP and AX2 GFP-DdN-NKAP as compared to AX2. AX2 cells began to stream within 8 h and formed large elaborately branched streams by 10 h and completed aggregation by approximately 12 h with a compact aggregation center. In contrast, GFP-DdNKAP cells did not initiate aggregation until 10 h but then streaming was normal with appreciable branching whereas GFP-DdN-NKAP cells showed premature stream formation already at 6 h. By 8 h large streams had formed, and by 10 h aggregation was completed (Fig 7E). The GFP-DdNKAP cells developed into normal fruiting bodies, although they exhibited a delay also in development on a solid substratum.

### The chemotactic speed is increased in GFP-DdN-NKAP cells

**Fig 8:**
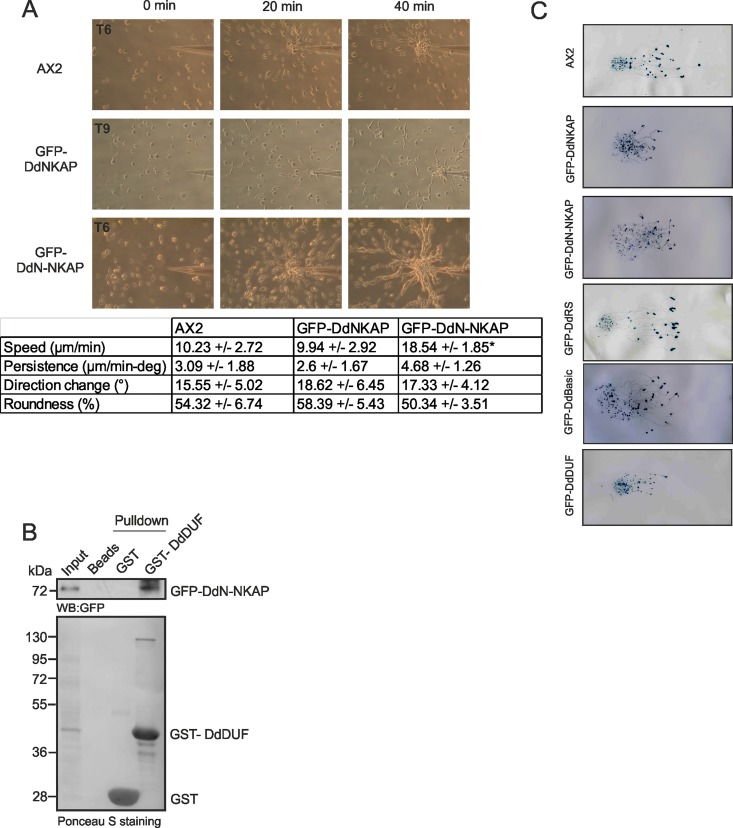
Motility analysis of GFP-DdNKAP mutants. (A) Chemotactic motility. Growth-phase cells were harvested, washed and developed for 6 h (AX2, GFP-DdN-NKAP) or 9 h (GFP-DdNKAP) in shaking suspension under starvation conditions to render them aggregation competent before they were challenged with cAMP in a chemotaxis assay. Time-lapse images were captured and processed. In the table the data for speed, persistence, direction change and roundness are given. Cells were recorded, and after tracing of the cells, the centroid of the cells was determined by computer-assisted analysis (DIAS). This allows calculations of speed, roundness (ratio of the long and short axis of the cell), and direction change. Persistence is a measure of movement in the direction of the path; direction change represents the average change of angle between frames in the direction of movement. The data shown are derived from five independent experiments. The data from 10 cells each were used for statistic evaluation. The difference in speed of AX2 and GFP-DdNKAP was significant (*p<0.01). (B) GST tagged DdDUF pull down of GFP-DdN-NKAP recognized by mAb GFP K184-3. GST-DdDUF and GST (control) were visualized by Ponceau S staining. (C) Wild type AX2 and DdNKAP mutant cells were harvested at the log phase of growth and resuspended at 1 x 10^8^ cells/ml and 10 μl containing 1x10^6^ cells were placed at the center of water agar plates. The plates were then placed in a dark box containing a slit of 3 mm and incubated at 21°C for 48 h. The slugs were transferred to nitrocellulose membranes and stained with amido black and studied for their movement towards the light source which was located on the right.

*D*. *discoideum* cells are highly motile. Furthermore, they can sense exogenous signals like cAMP which is secreted during development and migrate in an oriented fashion towards the chemotactic agent. For the analysis of single cell motility and behaviour in response to a cAMP stimulus, aggregation competent cells were allowed to migrate towards a capillary filled with 0.1 mM cAMP and time-lapse image series were captured to generate migration paths and calculate various parameters like speed, persistence, directionality and direction change. Within seconds of exposure to cAMP, the cells were able to orient themselves towards the chemoattractant source; they moved towards the pipette and organized themselves into streams (S1, S2 and S3 Movies). AX2 cells were polarized, formed streams and migrated at a speed of 10.23 +/- 2.72 μm/min. GFP-DdN-NKAP cells showed similar shape changes, however the speed was significantly increased with 18.54 +/- 1.85 μm/min. GFP-DdNKAP cells did not move towards the source of cAMP at the t6, t7 and t8 h time point but migrated with a speed of 9.94 +/- 2.92 μm/min at the t9 h time point confirming the developmental delay of this mutant (Fig 8A). All other parameters, persistence, direction change and roundness did not significantly differ in the strains (Table in Fig 8A). From the data it appears that overexpression of GFP-DdNKAP has an inhibitory effect on development whereas removal of the DdDUF domain in GFP-DdN-NKAP releases the inhibitory effect. This suggested to us an interaction between both parts of the protein. We therefore used GST-DdDUF and GFP-DdN-NKAP pulldown assays in order to probe the DdN-NKAP and DdDUF interaction and found that GST-DdDUF efficiently precipitated GFP-DdN-NKAP (Fig 8B).

Chemotactic aggregation of starving *Dictyostelium* cells leads to formation of a motile, multicellular organism, the slug, whose anterior tip controls its phototactic and thermotactic behaviour [38]. We therefore studied phototaxis in DdNKAP overexpressors. GFP-DdRS and GFP-DdBasic positive slugs were capable of phototaxing in a highly directed manner towards the light source like AX2. GFP-DdNKAP, GFP-DdN-NKAP and GFP-DdDUF strains were able to form migrating slugs but they differed in comparison to wild type slugs as they did not migrate as far as AX2 slugs in the same period of time (Fig 8C). From these data it appears that DdNKAP impairs various aspects of the *D*. *discoideum* life cycle.

## Discussion

In recent years, the SR proteins have been extensively characterized as a family of multifunctional proteins. They are critically involved in splicing, RNA export, translation and cell cycle control [39]. Since DdNKAP is a SR related protein and an ortholog of human NKAP which has been shown to be a none-core spliceosomal protein found in lower abundance at the spliceosome [10], we hypothesized that DdNKAP overexpression would affect the splicing process. However, we did not find appreciable alterations in constitutive splicing in the DdNKAP overexpressors. Instead, we observed a global disturbance of abundance patterns of many transcripts in microarray and RNAseq data. Our analysis of the cells further suggests that an overexpression of DdNKAP can affect processes driven by the action of differential levels of transcripts at certain developmental times such as during aggregation, chemotaxis and phototaxis. The RNAseq data could be related to developmental data available from mutant analysis as e.g. delayed aggregation was found in the srsA (Starvation responsive small gene A) overexpressors [40]. For this gene we found higher transcript levels, whereas the grlE (metabotrobic Glutamate Receptor-Like) transcript levels were reduced which is in agreement with delayed aggregation of the knockout strain [41]. It has been proposed that non-core proteins can link the spliceosome to other machineries such as transcription factors and therefore have the potential to affect cellular processes [42,43].

For efficient gene expression various machineries are functionally coupled *in vivo* from transcription to RNA processing to RNA export [44,45]. Prp19 functions in diverse processes including transcription, splicing, DNA repair, protein degradation and lipid droplet formation [35]. Similarly, mammalian NKAP is a multifunctional protein and has been previously described as transcriptional repressor and spliceosomal component [3,4]. Moreover, *Arabidopsis* MAS2 is a key player in the regulation of rRNA synthesis in plants [7]. Based on the well characterized role of Prp19 in splicing, the physical interaction of DdNKAP and Prp19 further helps to explain the participation of DdNKAP in RNA biogenesis. Prp19 exhibits E3 ubiquitin ligase activity and can directly interact with DdNKAP *in vitro*. Prp19 may therefore target DdNKAP for ubiquitination, a modification that can have a number of consequences such as an influence on protein stability, conformation, activity or localization [46]. Prp19 is essential for splicing but is not a constituent of any spliceosomal snRNPs [47]. Interestingly, our CLIP data revealed that DdNKAP interacts with U1 and U2 snRNAs. Therefore, the DdNKAP-Prp19 interaction could provide a bridge between the Prp19 complex and snRNPs. Although our report puts more emphasis on the role of DdNKAP in RNA binding, we cannot rule out the possibility that DdNKAP could play a potential role in transcriptional control. Prp19 functions in transcription elongation that is essential for TREX occupancy, a complex that couples transcription to mRNP export [48]. The significance of the DdNKAP-Prp19 interaction could then be in transcription control. However, we could not test this possibility as we were not successful in generating knockout and knockdown mutants. We assume that the loss of DdNKAP leads to a lethal phenotype. In *Arabidopsis*, insertional disruption of the coding region of MAS2 caused embryonic lethality [7]. Lethal phenotypes have also been described for NKAP knockouts in *C*. *elegans*, *D*. *melanogaster*, and mouse [2,4,8].

Beyond the DdNKAP-Prp19 interaction, our co-immunoprecipitation studies revealed that DdNKAP interacts with ribosomal subunit proteins. Ribosomal proteins are often found in immunoprecipitations and have been proposed to bind nonspecifically to proteins. However, LCMS analysis showed a higher number of hits for ribosomal proteins which suggests a specific interaction. Interestingly, in *Arabidopsis* splicing and ribosome biogenesis proteins have been identified as interactors of MAS2 in yeast two-hybrid assays. Additionally, the suppressor *mas2* mutations modified the splicing and/or translational efficiency [7]. Some of our results in the GFP-DdNKAP mutant relate to modified translational efficiency. First, we observed down regulation of transcripts for ribosomal proteins. Second, we observed up regulation of transcripts for ubiquitin and stress response In *D*. *discoideum*, inhibition of protein synthesis by cycloheximide leads to strong accumulation of ubiquitin transcripts [49]. In other words, the higher levels of ubiquitin transcripts could also result from an inhibition of translation in the GFP-DdNKAP cells. The modified translational efficiency in the DdNKAP mutant could be explained as follows. The unprocessed pre-mRNAs/pre-rRNAs accumulated due to aberrant splicing in the GFP-DdNKAP mutant. In general, stress response genes could also be activated due to the overexpression of the fusion protein. But we cannot deny the possibility of activation of stress response genes due to translation inhibition/modification. This is an interesting finding and should be addressed further. Through RNAseq analysis we found that the overexpression of DdNKAP had a minor effect on the splicing process. Furthermore, qRT-PCR revealed that rRNAs were upregulated in DdNKAP overexpressors which could be due to the accumulation of unprocessed pre-rRNAs and this possibility should be tested in the future. Similarly, an accumulation of unprocessed pre-rRNAs was observed in MAS2 mutant, suggesting MAS2’s role in the regulation of the translation of aberrant or unspliced mRNAs, by regulating their nuclear export [7].

We found that DdNKAP interacts and partially colocalize with the nuclear envelope SUN1 protein. Mammalian SUN1 mediates mRNA export through its association with mRNP complexes via a direct interaction with hnRNPs, nuclear RNA export factor 1 (NXF1) and NUP153 [50]. An impact of DdNKAP on mRNA export might therefore also be relevant for our findings. Additionally, in mammals, the DUF926 domain is present in two proteins, NKAP and NKAP-L. The nuclear speckle localizing NKAP acts as transcriptional repressor and is also a spliceosomal component whereas the uncharacterized protein NKAP-L localizes to the nucleolus (our unpublished data). A role for NKAP-L in rRNA transcription and/or rRNA biogenesis is also plausible. *D*. *discoideum* contains a single DUF926 containing protein, DdNKAP, which might localize to nucleoli and may perform the functions of NKAP and NKAP-L i.e. have roles in transcription/mRNA biogenesis/rRNA biogenesis. Recent reports identified RbdB as an essential component of miRNA processing in *Dictyostelium discoideum*. Two putative domains were identified in RbdB, a double stranded RNA binding domain (dsRBD) and a proline rich site. dsRBD domains in *Dictyostelium* are functionally conserved and show functional redundancy as the dsRBDs of DrnB and RdbB can be functionally exchanged [51]. RbdB localizes to nuclei with distinct foci at nucleoli and ablation of rbdb results in loss of miRNAs [52]. Based on the localization of DdNKAP in a punctate pattern in nuclei and its ability of interact with RNA, the likelihood of DdNKAP to bind and process miRNAs must be addressed further.

Finally, with regard to developmental phenotypes, mutants overexpressing full length DdNKAP and DdN-NKAP overexpressor showed opposite phenotypes in aggregation, streaming and chemotaxis. In agreement with these results, we found an opposite expression profile of several genes and rRNAs in GFP-DdNKAP and GFP-DdN-NKAP overexpressors. Similarly, differing localizations were observed upon ectopic expression of NKAP and the N-terminus of NKAP in mammalian cells [3]. We also confirmed the interaction of the N-terminal domain and DdDUF926 domain biochemically. Based on these results we propose that the DdDUF926 domain of DdNKAP acts as a negative regulator of the N-terminal domain.

In conclusion, we present the first characterization of an SR protein in *D*. *discoideum*, namely DdNKAP. DdNKAP is the single DUF926 domain containing protein present in *D*. *discoideum*. The functional similarity between Arabidopsis MAS2, human NKAP and DdNKAP is very high. Taken together it seems that NKAPs are multifunctional proteins and function in the regulation of transcription, translation and RNA export.

## Supporting Information

S1 Supporting Information Supporting methods (DOCX)

S1 Fig Cytosolic and nuclear fractionation. (A) Cytosolic and nuclear fractionation of GFP-DdNKAP and GFP-DdNKAP expressing cells. GFP tagged proteins were visualized by mAb K3-184-2. α-Tubulin was used as control. C: cytosolic, N: nuclear fraction. (B) Autoradiograph of control and cross-linked DdNKAP-RNA complexes separated by denaturing gel electrophoresis (4–12% acrylamide) and transferred to a membrane. The UV treated and untreated samples were subjected to partial RNA digestion using low or high concentration of RNase T1. (C) Confirmation of increased expression of DGAP1 and Cortexillin I by western blot with DGAP1 and Cortexillin I antibodies. α-Tubulin detected by rat mAb YL1/2 was used as control. (D) Densitometric analysis of DGAP1 and Cortexillin I levels. The bar graph shows fold increase of DGAP1 and Cortexillin I in DdNKAP overexpressors.
(TIF)

S2 Fig Generation of a knockout vector. (A) The targeting vector with a blasticidin resistence cassette was generated by cloning a 476 bp long arm homologous to the 5´ region of the DdNKAP gene to the left of the cassette and a 563 bp long arm homologues to the 3´ region to the right of the cassette. (B) DdNKAP knockdown vector. The shorter PCR fragment contains 417 base pairs, whereas the longer one contains 511 base pairs. The two PCR products were ligated in a tail-to-tail orientation and subcloned under the Actin-15 promoter to produce the stem loop structure.
(TIF)

S1 Table Primers used for qRT-PCR experiments. (DOCX)

S2 Table Identification of interaction partners of Sun-1. GFP-Sun-1 was used to identify interacting partners. AX2 cells expressing GFP-Sun-1 were lysed and the supernatant was incubated with GFP antibodies bound to protein A Sepharose beads. The samples were resolved in SDS polyacrylamide gels (12% acrylamide) and individual bands cut from the gel. The protein bands analyzed and identified by MALDI-MS are listed.
(DOCX)

S3 Table Identification of interaction partners of DdNKAP. Axenically growing GFP DdNKAP expressing AX2 cells were harvested, washed twice with Soerensen phosphate buffer, pH 6.0, lysed and the particulate material was removed by centrifugation. The supernatant was used for immunoprecipitation with the anti-GFP mAb K3-184-2. The co-immunoprecipitated proteins were resolved in SDS polyacrylamide gels (12% acrylamide) and individual bands cut from the gel. The protein bands analyzed and identified by MALDI-MS are listed. The blue and red colours indicate RNA binding proteins and ribosomal proteins, respectively.
(DOCX)

S4 Table Mapping information. DdNKAP1, DdNKAP2, DdNKAP3, DdNKAP4 are replicates.
(DOCX)

S5 Table The uniquely identified genes along with their mapped genomic features in CLIPseq. The CLIP raw reads were aligned to the *D*. *discoideum* genome sequence (http://dictybase.org/) using Bowtie2. We counted reads associated with genomic features with the tophat2 program.
(XLSX)

S6 Table Significantly up- and down-regulated genes of GFP-DdNKAP expressing AX2 cells found by microarray analysis. Differentially regulated genes were identified using the SAM program. Only those genes are listed that were in addition more than 1.5 fold differentially regulated in comparison to AX2 cells. GeneID is the dictyBase (http://dictybase.org/) gene identification number. The description of the protein name was obtained from dictyBase. Proteins were functionally categorised using the extended categorization scheme for *D*. *discoideum* that is based on the yeast classification scheme.
(XLSX)

S7 Table Significantly up- and down-regulated genes of GFP-DdNKAP expressing AX2 cells found by RNAseq. Differentially regulated genes were identified using the DEseq package. GeneID is the dictyBase (http://dictybase.org/) gene identification number.
(XLSX)

S1 Movie Chemotaxis was performed with AX2. (AVI)

S2 Movie Chemotaxis was performed with GFP-DdNKAP expressing cells. (AVI)

S3 Movie Chemotaxis was performed with GFP-DdN-NKAP expressing cells. (AVI)
