# Supplementary material for: The C-Terminal SynMuv/DdDUF926 Domain Regulates the Function of the N-Terminal Domain of DdNKAP
Source: PLoS One. 2016 Dec 20;11(12):e0168617. doi: 10.1371/journal.pone.0168617 (PMC5173251; doi:10.1371/journal.pone.0168617)
Supplement: S1 Supporting Information — (DOCX) [file pone.0168617.s001.docx]

**S1 Supporting Information**

**Supporting methods**

**Generation of DdNKAP knockout and knockdown vectors**

A DdNKAP knockout vector was generated by cloning a 476-bp (4-480 nt) SalI/HindIII fragment of the 5′ end upstream and a 563-bp (817-1380 nt) PstI/BamHI downstream fragment of the coding region into the corresponding sites of pLPBLP containing the blasticidin resistance cassette. The resulting vector was cleaved with SalI and BamHI and transformed into wild type cells. At least 50 clones were analyzed by PCR.

Construction of the RNAi-encoding plasmid for DdNKAP knockdown was done by cloning two partially redundant fragments. The 417 bp fragment in sense orientation was cloned into BamHI/SalI-digested pUC18. The second fragment of 538 bp was cloned in reverse orientation into the generated pUC18 plasmid after digestion with PstI/SalI. Finally the fused fragments were subcloned into PstI/BamHI-digested pDNeoII [1]. The resulting plasmid was transformed into AX2 wild type cells. At least 50 clones were tested by western blot analysis using DdNKAP antibodies.

**Cytosolic nuclear fractionation**

Vegetatively growing cells (1 x 10^7^) washed with Soerensen phosphate buffer, pH 6.0, twice and suspended in TMS buffer (50 mM Tris/HCl, pH 7.4, 100 mM NaCl, 5 mM MgCl_2_, 250 mM Sucrose, 1 mM EDTA, 1 mM EGTA). Cells were lysed by passing through Nuclepore membrane (pore size 5µm, Whatman). Nuclei were collected after cell lysis by spinning for 5 min at 4000 g.

**References:**

1. Witke W, Nellen W, Noegel A. Homologous recombination in the Dictyostelium alpha-actinin gene leads to an altered mRNA and lack of the protein. EMBO J. 1987; 6: 4143–4148.
