# Supplementary figures and images for: The C-Terminal SynMuv/DdDUF926 Domain Regulates the Function of the N-Terminal Domain of DdNKAP
Source: PLoS One. 2016 Dec 20;11(12):e0168617. doi: 10.1371/journal.pone.0168617 (PMC5173251; doi:10.1371/journal.pone.0168617)

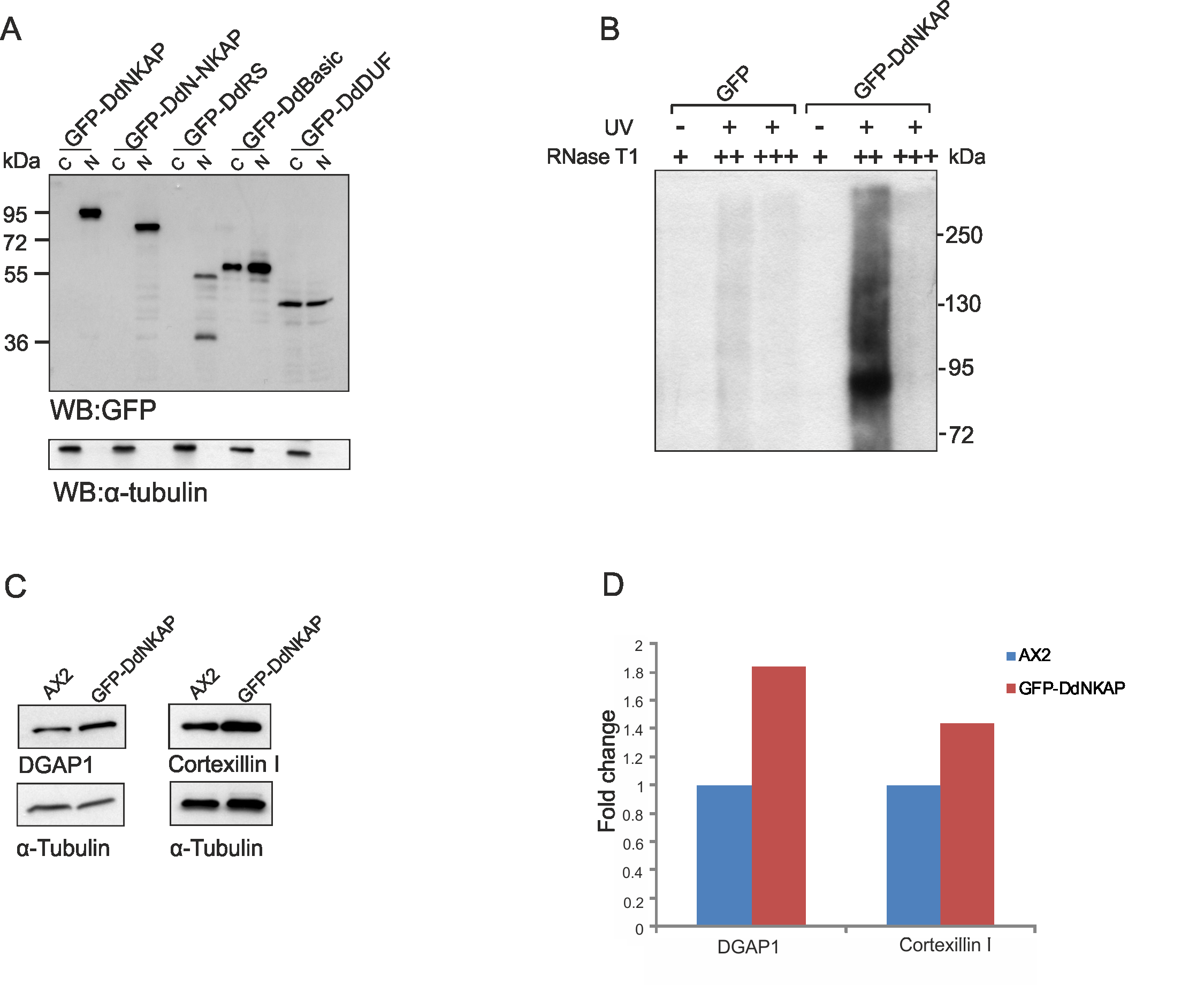

Supplement: S1 Fig — (A) Cytosolic and nuclear fractionation of GFP-DdNKAP and GFP-DdNKAP expressing cells. GFP tagged proteins were visualized by mAb K3-184-2. α-Tubulin was used as control. C: cytosolic, N: nuclear fraction. (B) Autoradiograph of control and cross-linked DdNKAP-RNA complexes separated by denaturing gel electrophoresis (4–12% acrylamide) and transferred to a membrane. The UV treated and untreated samples were subjected to partial RNA digestion using low or high concentration of RNase T1. (C) Confirmation of increased expression of DGAP1 and Cortexillin I by western blot with DGAP1 and Cortexillin I antibodies. α-Tubulin detected by rat mAb YL1/2 was used as control. (D) Densitometric analysis of DGAP1 and Cortexillin I levels. The bar graph shows fold increase of DGAP1 and Cortexillin I in DdNKAP overexpressors. (TIF) [file pone.0168617.s002.tif]

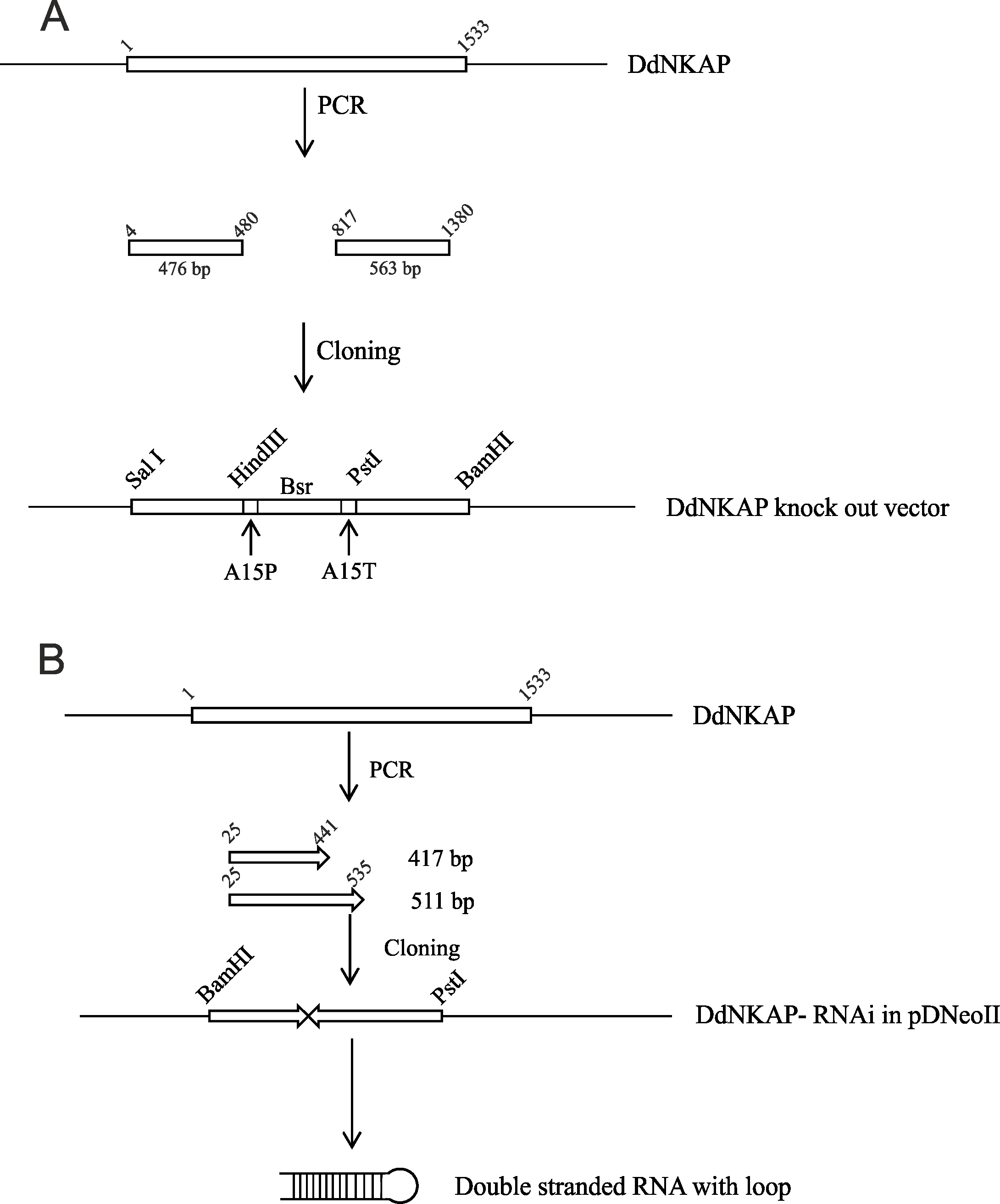

Supplement: S2 Fig — (A) The targeting vector with a blasticidin resistence cassette was generated by cloning a 476 bp long arm homologous to the 5´ region of the DdNKAP gene to the left of the cassette and a 563 bp long arm homologues to the 3´ region to the right of the cassette. (B) DdNKAP knockdown vector. The shorter PCR fragment contains 417 base pairs, whereas the longer one contains 511 base pairs. The two PCR products were ligated in a tail-to-tail orientation and subcloned under the Actin-15 promoter to produce the stem loop structure. (TIF) [file pone.0168617.s003.tif]
