# Supplementary material for: The C-Terminal SynMuv/DdDUF926 Domain Regulates the Function of the N-Terminal Domain of DdNKAP
Source: PLoS One. 2016 Dec 20;11(12):e0168617. doi: 10.1371/journal.pone.0168617 (PMC5173251; doi:10.1371/journal.pone.0168617)
Supplement: S1 Table — (DOCX) [file pone.0168617.s004.docx]

| 26S_rRNA FW | 5´GAACAACCTAGTAGCTGGTTCCTTC 3´ |
| --- | --- |
| 26S_rRNARV | 5´CTCCTATTTTTCATTGCACATGTGC3´ |
| 17S_rRNA FW | 5´GTGACAATAAATATCAATACCTATC3´ |
| 17S_rRNA RV | 5´CGGTGTCGATTTAACCACGAAGTGG3´ |
| RPL15 FW | 5´GTCCACAAACATAGAGAATTAAG 3´ |
| RPL15 RV | 5´TTATCTGTATCTGTGGAAAACG 3´ |
| RPL35 FW | 5´CATCAAGCAAGATCCCAACTGAT 3´ |
| RPL35 RV | 5´TTAGGCTTTAACAGCGAAAACTC 3´ |
| RPL9 FW | 5´GGTGTCATGGAAGAATCAAAGAA 3´ |
| RPL9 RV | 5´ATGGATTGATTTGGCAAGACCAT 3´ |
| Mhc1 FW | 5´CCAGAATCATTCAACTACTTAAA 3´ |
| Mhc1 RV | 5´TTCTTCTTGTGAGAAACCAACAA 3´ |
| WarA FW | 5´GCCTCTGTCATTGATTCAAACAA 3´ |
| WarA RV | 5´ATCAATGTCAATGGCATTCATAT 3´ |
| Gata FW | 5´CCACCATCATCAAAAACAAAAGG 3´ |
| Gata RV | 5´GATATCATCAGTGGTTGTAGTAG 3´ |

**S1 Table.** **Primers used for qRT-PCR experiments.**
