# Supplementary material for: The C-Terminal SynMuv/DdDUF926 Domain Regulates the Function of the N-Terminal Domain of DdNKAP
Source: PLoS One. 2016 Dec 20;11(12):e0168617. doi: 10.1371/journal.pone.0168617 (PMC5173251; doi:10.1371/journal.pone.0168617)
Supplement: S2 Table — GFP-Sun-1 was used to identify interacting partners. AX2 cells expressing GFP-Sun-1 were lysed and the supernatant was incubated with GFP antibodies bound to protein A Sepharose beads. The samples were resolved in SDS polyacrylamide gels (12% acrylamide) and individual bands cut from the gel. The protein bands analyzed and identified by MALDI-MS are listed. (DOCX) [file pone.0168617.s005.docx]

| Gene ID | Gene Product | Peptide coverage (%) | Number of Peptides |
| --- | --- | --- | --- |
| DDB0220460 | actin | 42 | 16 |
| DDB0191444 | myosin II heavy chain | 38.61 | 76 |
| DDB0191262 | DEAD/DEAH box helicase domain-containing protein | 30.63 | 10 |
| DDB0306239 | DUF926 family protein DdNKAP | 21.18 | 14 |
| DDB0231241 | 60S ribosomal protein L4 | 11.3 | 3 |
| DDB0191318 | cystathionine gamma-lyase | 10.8 | 3 |
| DDB0215012 | cathepsin D | 7.57 | 2 |
| DDB0231294 | isocitrate dehydrogenase | 5.3 | 2 |
| DDB0191135 | elongation factor 1a | 2 | 1 |
| DDB0191363 | elongation factor 2 | 1.3 | 1 |
| DDB0233434 | SNF2-related domain-containing protein | 1 | 1 |

**S2 Table. Identification of interaction partners of Sun-1**
