# Supplementary material for: The C-Terminal SynMuv/DdDUF926 Domain Regulates the Function of the N-Terminal Domain of DdNKAP
Source: PLoS One. 2016 Dec 20;11(12):e0168617. doi: 10.1371/journal.pone.0168617 (PMC5173251; doi:10.1371/journal.pone.0168617)
Supplement: S3 Table — Axenically growing GFP DdNKAP expressing AX2 cells were harvested, washed twice with Soerensen phosphate buffer, pH 6.0, lysed and the particulate material was removed by centrifugation. The supernatant was used for immunoprecipitation with the anti-GFP mAb K3-184-2. The co-immunoprecipitated proteins were resolved in SDS polyacrylamide gels (12% acrylamide) and individual bands cut from the gel. The protein bands analyzed and identified by MALDI-MS are listed. The blue and red colours indicate RNA binding proteins and ribosomal proteins, respectively. (DOCX) [file pone.0168617.s006.docx]

**S3 Table. Identification of interaction partners of DdNKAP**

| Gene ID | Gene Product | Peptide coverage (%) | Number of Peptides |
| --- | --- | --- | --- |
| DDB0230021 | rps3a | 62.6 | 22 |
| DDB0201558 | ancA | 57 | 22 |
| DDB0304484 | hyaluronan/mRNA binding family protein | 49.8 | 10 |
| DDB0185063 | rps4 | 48.3 | 17 |
| DDB0191525 | smlA | 46.6 | 20 |
| DDB0231242 | rpl5 | 46.2 | 10 |
| DDB0219977 | rplP0, ribosomal acidic phosphoprotein P0 | 45.2 | 14 |
| DDB0233340 | RNA-binding region RNP-1 domain-containing protein | 44.7 | 9 |
| DDB0231339 | rpl7a | 41.8 | 18 |
| DDB0230023 | rps6 | 41.5 | 9 |
| DDB0215391 | rps2 | 31.3 | 8 |
| DDB0267046 | Fbl, fibrillarin | 28.1 | 8 |
| DDB0230193 | pdhA, Pyruvate DeHydrogenase | 27.6 | 11 |
| DDB0230016 | rpsA | 25.7 | 4 |
| DDB0231242 | rpl5 | 25.4 | 12 |
| DDB0237882 | prsA, phosphoribosyl pyrophosphate synthetase | 24.6 | 4 |
| DDB0231494 | RNA-binding region RNP-1 domain-containing protein | 24.1 | 6 |
| DDB0185227 | V-ATPase subunit D vatD-1 | 20.8 | 7 |
| DDB0216215 | V-ATPase subunit M vatM | 19 | 14 |
| DDB0237963 | pdx1, putative pyridoxal biosynthesis protein PDX1 | 16.7 | 3 |
| DDB0214925 | ubqB, ubiquitin/ribosomal protein L40 fusion protein | 16.4 | 5 |
| DDB0229442 | pdhB | 14.9 | 4 |
| DDB0233910 | eif3I, Eukaryotic translation Initiation Factor 3 subunit I | 14.5 | 3 |
| DDB0266395 | Bysl, bystin, processing of 20S pre-rRNA precursor and biogenesis of 40S ribosomal subunits | 11.9 | 4 |
| DDB0231063 | rps24 | 11.9 | 2 |
| DDB0215400 | dscA-1 | 11.1 | 2 |
| DDB0229992 | mcfZ | 10.6 | 4 |
| DDB0230188 | mdhB, malate dehydrogenase | 9.8 | 2 |
| DDB0231332 | hisS, histidyl-tRNA synthetase | 9.6 | 3 |
| DDB0233121 | prp19 (Pre-mRNA Processing) | 8.8 | 3 |
| DDB0201638 | rpl27a | 8.8 | 1 |
| DDB0237965 | smt1, Sterol MethylTransferase | 7.6 | 2 |
| DDB0230186 | mdhA, malate dehydrogenase | 7.4 | 2 |
| DDB0308488 | 4-alpha-glucanotransferase | 6.2 | 5 |
| DDB0233888 | mcfN | 6 | 2 |
| DDB0233701 | cpsf4, Cleavage and Polyadenylation Specificity Factor | 5.9 | 2 |
| DDB0237782 | atp5C1 | 5.7 | 1 |
| DDB0214941 | similar to smlA | 5.7 | 1 |
| DDB0215387 | pdhC (dihydrolipoamide acetyltransferase) | 4.6 | 2 |
| DDB0266779 | Denr, density-regulated protein, translation initiation | 4.5 | 1 |
| DDB0216346 | csnk2b, CaSeiN Kinase II subunit Beta | 4.5 | 1 |
| DDB0216283 | gtf2b, Global Transcription Factor II B | 4.3 | 1 |
| DDB0232063 | phbA, ProHiBitin | 4.1 | 1 |
| DDB0214935 | arcB | 4.1 | 1 |
| DDB0229901 | dynamin like | 4 | 3 |
| DDB0238285 | mrpl3, ribosomal protein L3, mitochondrial | 4 | 1 |
| DDB0191205 | vps26, Vacuolar Protein Sorting | 3.7 | 1 |
| DDB0304712 | putative mitochondrial import inner membrane translocase subunit | 3.5 | 1 |
| DDB0238021 | contains a putative N-terminal signal sequence and a C-terminal transmembrane domain | 3.5 | 1 |
| DDB0191246 | gpaE, Galpha5 | 3.2 | 1 |
| DDB0231399 | tom40, mitochondrial porin | 3.2 | 1 |
| DDB0191255 | gpaA, *Galpha1* | 3.1 | 1 |
| DDB0216232 | Lpd, dihydroLiPoyl Dehydrogenase | 2.7 | 1 |
| DDB0231308 | aspS1 (aspartate-tRNA synthetase) | 2.6 | 1 |
| DDB0216293 | rpc1, RNA Polymerase III | 2.4 | 2 |
| DDB0229442 | pdhB, Pyruvate DeHydrogenase Beta subunit | 2.2 | 1 |
| DDB0233341 | pigN, PhosphatidylInositol Glycan | 1.5 | 1 |
| DDB0191363 | elongation factor 2 (EF2) | 1.1 | 1 |
| DDB0233083 | SNF2-related domain-containing protein | 1 | 1 |
