# Supplementary material for: The C-Terminal SynMuv/DdDUF926 Domain Regulates the Function of the N-Terminal Domain of DdNKAP
Source: PLoS One. 2016 Dec 20;11(12):e0168617. doi: 10.1371/journal.pone.0168617 (PMC5173251; doi:10.1371/journal.pone.0168617)
Supplement: S4 Table — DdNKAP1, DdNKAP2, DdNKAP3, DdNKAP4 are replicates. (DOCX) [file pone.0168617.s007.docx]

**S4 Table**: **Mapping information.**

|  | DdNKAP1 | DdNKAP2 | DdNKAP3 | DdNKAP4 |
| --- | --- | --- | --- | --- |
| Reads Mapped | 4215985 | 269384 | 33190618 | 32294718 |
| Reads properly paired | 3922508 | 180528 | 32201264 | 30703178 |
| Total reads | 67007478 | | | |
